# Reply to: Admission factors associated with intensive care unit readmission in critically ill oncohematological patients: a retrospective cohort study

**DOI:** 10.5935/0103-507X.20160062

**Published:** 2016

**Authors:** Cinthia Mendes Rodrigues, Ellen Maria Campos Pires, Jorge Patrick Oliveira Feliciano, Jose Mauro Vieira Jr., Leandro Utino Taniguchi

**Affiliations:** Research and Education Institute (IEP), Hospital Sírio-Libanês - São Paulo (SP), Brazil.; Emergency Medicine Discipline, Hospital das Clínicas, Faculdade de Medicina, Universidade de São Paulo, São Paulo (SP), Brazil.

We would like to thank you for the interest in our study about admission factors associated with later readmission to the intensive care unit (ICU) in an oncohematological cohort.^(1)^ Aydoğdu and Esquinas correctly acknowledged that only evaluating ICU admission factors limited our analysis. Unfortunately, we evaluated an administrative database, which only had some physiological information related to the first 24 hours of ICU stay (due to prognostic scores calculation) but lacked data about the conditions at ICU discharge. This is a limitation, as stated before in our article. We also share the impression that data at ICU discharge might be a better discriminator of later unexpected events (such as death or readmission). In fact, we are now performing this study in our unit, and results are expected soon. Nevertheless, it should also be acknowledged that Hosein et al.^(2)^ recently published a systematic review of tools [including the Stability and Workload Index for Transfer (SWIFT) score^(3)^] trying to predict readmission after ICU discharge. One of their main conclusions is that, although many scores have been published, none of them has clearly demonstrated improvement of clinical outcomes.^(2)^

Finally, to clarify some issues, we considered "mechanical ventilation" only as the use of invasive mechanical ventilation. Our database does not have information about infection-related events regarding opportunistic agents. The median length of first ICU stay in the non-readmission group was 2 [1 - 3] days versus 3 [2 - 5] days in the readmission group (p < 0.001 using the Mann-Whitney test). The inclusion of this independent factor in our logistic model did not alter our results.

Cinthia Mendes Rodrigues, Ellen Maria Campos Pires, Jorge Patrick Oliveira Feliciano and Jose Mauro Vieira Jr.

Instituto de Ensino e Pesquisa, Hospital Sírio-Libanês - São Paulo (SP), Brazil.

Leandro Utino Taniguchi

Instituto de Ensino e Pesquisa, Hospital Sírio-Libanês - São Paulo (SP), Brazil and Emergency Medicine Discipline, Hospital das Clínicas, Faculdade de Medicina, Universidade de São Paulo, São Paulo (SP), Brazil.
